# Anti-Cancer Activities of Diterpenoids Derived from *Euphorbia fischeriana* Steud

**DOI:** 10.3390/molecules23020387

**Published:** 2018-02-11

**Authors:** Baiyu Jian, Hao Zhang, Cuicui Han, Jicheng Liu

**Affiliations:** 1 Graduate School of Heilongjiang University of Chinese Medicine, Heilongjiang University of Chinese Medicine, Harbin 150040, China; abcaaa1@126.com; 2 Research Institute of Medicine and Pharmacy, Qiqihar Medical University, Qiqihar 161006, China; zhang101hao@aliyun.com; 3 College of Pharmacy, Qiqihar Medical University, Qiqihar 161006, China; hancuicui-1234@163.com

**Keywords:** diterpenoids, *Euphorbia fischeriana* Steud, anticancer activity, molecular mechanism

## Abstract

*Euphorbia fischeriana* Steud is an essential oriental folk medicine used for healing cancer, edema and tuberculosis. Recently, its anticancer activitity has attracted more attention. A volume of research has indicated that diterpenoids are the major anticancer active constituents from this medicinal herb. In this review, we aimed to provide a summary of the promising anticancer diterpenoids from this plant; many diterpenoids mentioned in this article are newly discovered diterpenoids. According to the carbon skeleton and substituents, they can be classified into eight subtypes: *ent*-abietane, daphnane, tigliane, ingenane, *ent*-atisane, *ent*-rosane, *ent*-kaurane, and lathyrane. Futhermore, their key anticancer mechanisms and protein targets of these compounds will be discussed. These natural diterpenoids could provide a reservoir for drug discovery.

## 1. Introduction

Cancer is a dreaded disease that ranks as the second-leading cause of death worldwide [1]. Systematic research proves that abnormal cell proliferation and metastasis are the two main characteristics of cancer development. Treatments for cancer include surgery, radiotherapy, and chemotherapy [2,3]. However, complete inhibition of tumor recurrence and metastasis is difficult to achieve using these methods [2,3]. Furthermore, the resistance of tumor cells to chemotherapeutic drugs is a significant problem, and urges the discovery of novel adjuvant therapies [1,4]. Traditional Chinese Medicine contains a lot of anticancer active substances and can provide considerable drug leads and candidates [5,6]. For this reason, researchers are always looking for inspiration from natural products.

*Euphorbia fischeriana* Steud, a perennial herbaceous plant of the family Euphorbiaceae, is mainly distributed in northern China [7,8]. Modern medical reseach has shown that the extracts and pure compounds of *E. fischeriana* exhibit a variety of pharmacological properties, incluing antitumor, antimicrobial, antiviral, immune enhancing, sedative and analgesic activities [7]. Among them, the research related to anticancer activity has attracted additional attention recently. Extracts of *E. fischeriana* have been proven to be effective against several types of cancer, including malignant melanoma, lewis lung carcinoma and ascitic hepatoma, in mice [7,9,10].

Chemical investigations of *E. fischeriana* have revealed the presence of diterpenoids, triterpenes, steroids and aromatic tannins [7]. Diterpenoids are the major components of *E. fischeriana*. They present variable skeletons with high oxidative functional moieties or acylated substituents [11,12,13]. These chemical and structural characteristics have attracted scientists interested in drug discovery research. Therefore, in recent years, study on the biological activities of diterpenoids from *E. fischeriana* has become a research focus. The body of research has noted that many of isolated diterpenoid compounds from this medicinal herb have cytotoxicities against a range of cancer cell types, and great strides have been taken in unraveling the mechanisms behind these effects. Diterpenoids are believed to be the major anticancer constituents of *E. fischeriana*.

In this paper, we aimed to provide a summary of the promising anticancer diterpenoids from this plant; many diterpenoids mentioned in this article are newly discovered diterpenoids. Futhermore, their key anticancer mechanisms and the protein targets of these compounds will be reported. These natural diterpenoids could provide a reservoir for drug discovery. 

## 2. Chemical Structure of Diterpenoids 

*E. fischeriana* produces a diversity of diterpenoids; researchers have found that approximately 24 diterpenoids have anticancer activities in *E. fischeriana*. Concerning the carbon skeleton and substituents at specific positions, these diterpenoids have been classified into eight subtypes; namely, *ent*-abietane (**1**, **2**, **3**, **4**, **15**, **16**, **17**), tigliane **(5**, **7**, **8**), daphnane (**6**), ingenane (**9**, **10**, **11**, **18**), *ent*-atisane (**12**, **13**, **19**, **20**) *ent*-rosane (**21**, **22**, **23**), *ent*-kaurane (**14**), and lathyrane (**24**).

**Figure 1:**
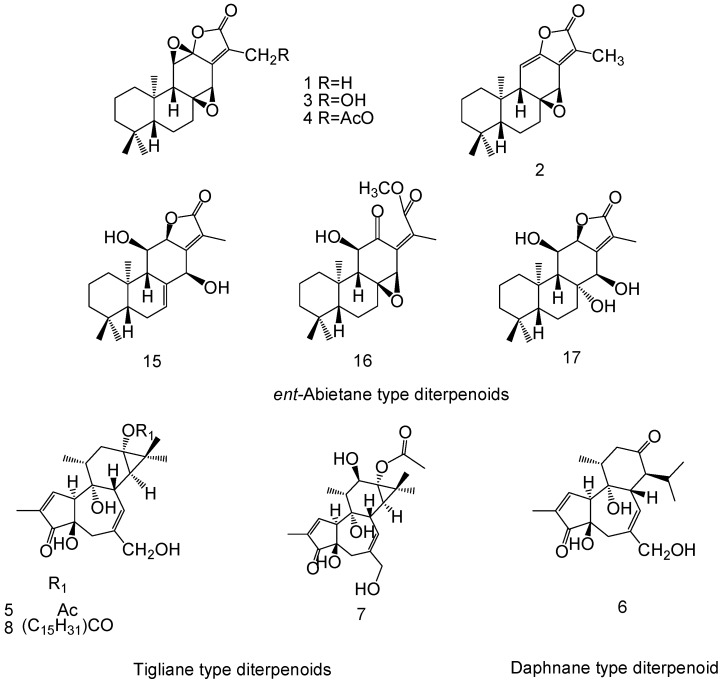
The chemical structures of *ent*-abietane-, tigliane- and daphnane-type diterpenoids derived from *E. fischeriana*.

**Figure 2:**
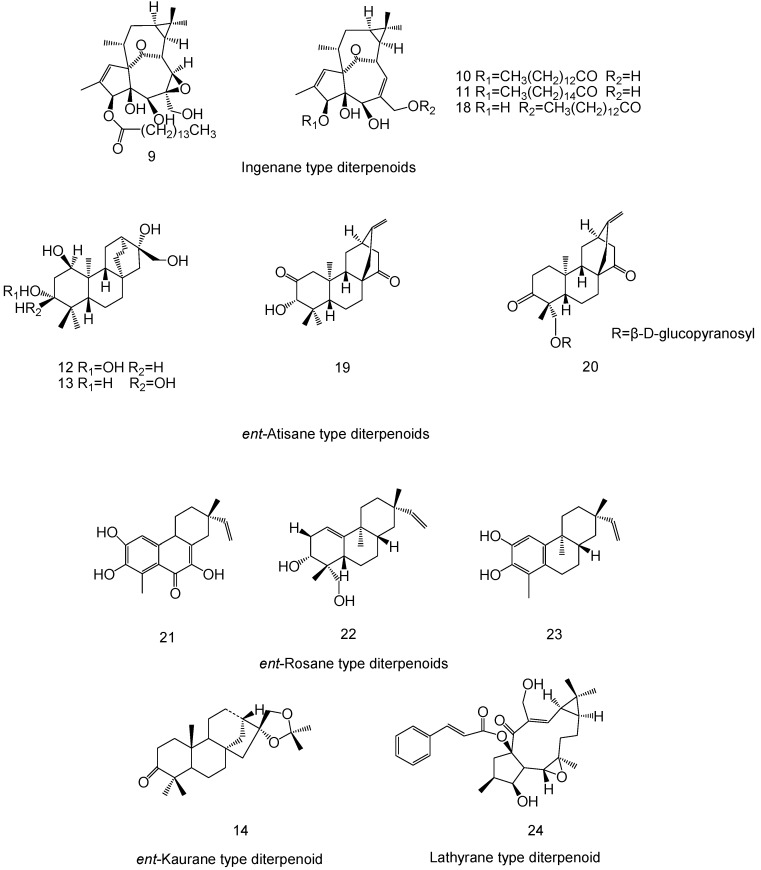
The chemical structures of ingenane-, *ent*-atisane-, *ent*-rosane-, *ent*-kaurane- and lathyrane-type diterpenoids derived from *E. fischeriana*.

Figure 1 and Figure 2 show the chemical structures of the diterpenoids derived from *E. fischeriana* with anticancer activities.

## 3. Anticancer Activities of Diterpenoids

In in vitro study, 14 diterpenoids (**1**–**14**) isolated from *E. fischeriana* have been found to inhibit the proliferation of several cancer cells with promising IC_50_ values. Their names, subtypes, cell toxicities and corresponding references are compiled in Table 1. 13 diterpenoids (**1**, **10**, **11**, **15**–**24**) showed inhibitory activities on the formation of mammospheres in human breast cancer MCF-7 cells [14,15]. This research result indicates the potential of these bioactive diterpenoids for further investigation of the action targeting cancer stem cells [14]. Their names, subtypes, and corresponding references are compiled in Table 2.

Several in vivo research studies have provided evidence supporting the anti-cancer activities of compound **1** and compound **8**. Here, we summarize detailed information about these studies in Table 3. The in vivo antitumor effects of other compounds need to be tested in future experiments.

## 4. The Anti-Cancer Mechanism of Diterpenoids 

### 4.1. Induction of Apoptosis

Apoptosis is an ordered and orchestrated cellular process that occurs in physiological and pathological conditions [33,34,35]. The process involves ordered morphological and biochemical events, including nuclear chromatin condensation, cell shrinkage, membrane blebbing, chromosomal DNA breakage and modulation of precise signaling circuitry [36,37]. Evading apoptosis and resisting cell death is one of the ten hallmarks of cancer [38]. Therefore, apoptosis is becoming a focus for oncology research. A hopeful field of anticancer strategies is applying medicine to start the tumor cell apotosis process [39,40].

Compound **1**, an *ent*-abietane type diterpenoid extracted from plants of the *E. fischeriana,* has been reported to exhibit promising anticancer activity by activating apoptosis in solid and liquid tumors, including human Leukemic, breast cancer and mouse melanoma [17,18,21,22,23]. At the molecular level, Jolkinolide B was found to inhibit JAK2/STAT3 pathway in human Leukemic HL-60 and THP-1 cells [21]. Compound **1** treatment led to downregulation of JAK2/STAT3 and bcl-2 and upregulation of Bax and cytosolic cytochrome c, thus triggering caspase-3, -8 and -9 activation-mediated apoptotic induction. On the other hand, compound **1** can interfere with PI3K/Akt pathways, leading to cancer cell apoptosis in MDA-MB-231 cells and Human Leukemic U937 cells [18,19]. In addition, a novel mechanistic finding showed that Jolkinolide B induced apoptosis in mouse melanoma B16F10 cells by altering glycolysis [23]. In the course of study, compound **1** was found to downregulate the mRNA expression of glucose transporter genes (Glut1, Glut3 and Glut4) and glycolysis-related kinase genes (Hk2 and Ldha), increase ROS level, and decrease the potential of the mitochondrial membrane, subsequently inducing tumor cells apoptosis in B16F10 cells [23]. Recent research has demonstrated that aerobic glycolysis is the main metabolic way by which most tumor cells produce ATP for growth and proliferation [23,41,42]. Therefore, inhibition of the glycolytic pathway may be a promising approach to inhibit cancer cell proliferation and induce cell apoptosis in tumor cells [23,43]. Compound **3** has also been shown to induce apoptosis in human cancer cells. The anticancer mechanism operates through inactivation of the JAK family kinases—JAK1, JAK2, and TYK2—by covalent cross-linking of the JAKs and blocking JAK/STAT3 signaling [25]. It is a promising anticancer drug candidate as a potent STAT3 signaling inhibitor [25]. Compound **4** has been reported as a novel type of NF-kB pathway inhibitor. It can keep IKK in its phosphorylated form irreversibly, and this effect leads to compound **4** effectively inhibiting tumor necrosis factor-A–induced NF-KB activation and inducing apoptosis of tumor cells [26]. It is another novel type of anticancer drug candidate [26]. All three diterpenoids belong to the abietane type. Compound **8**, a tigliane type diterpenoid extracted from this plant, has been found to induce apoptosis in BGC823 cells via caspase-3/caspase-9-dependent pathway [29]. In short, these diterpenoids modulate several signaling pathways, which results in apoptosis of tumor cells. The various mechanisms of Compounds **1**, **3**, **4**, **8** involved in inducing apoptosis are summarized in Table 4. 

### 4.2. Cell Cycle Arrest

Cell cycle deregulation is one of the important features of tumor cells. The abnormal expression and activity of cyclins, cyclin-dependent kinases and tumor suppressor proteins may directly affect cell cycle progression and tumorigenesis [44,45,46]. Some of the diterpenoids possess the ability to induce cell cycle arrest (Table 5). For instance, compound **1** can block cell cycles at G1 in human myeloid Leukemic cell K562 [20]. Likewise, compound **1** can cause cell cycle arrest of prostate cancer in the G1 phase [24]. Compound **8** can restrain cell cycle arrest at the G2-M checkpoint in gastric cancer through downregulation of cdc2/cyclin B, cyclin A and p-chk1 protein expression [29]. The effects of other diterpenoids from *E. fischeriana* on the blockage of cell cycles are an ongoing topic of research.

### 4.3. Inhibition of Metastasis 

Tumors possess the ability to transfer throughout the body and grow, a process known as metastasis, which is the leading cause of death from cancer. It is difficult to prevent of metastasis at present, since this process involves multiple stages, such as passing through the extracellular matrix, interaction with host lymphoid cells, and adhesion to basement membranes to form metastases. Fortunately many plant-derived compounds have been discovered to effectively suppress metastasis of tumor cells [47,48,49]. Compound **1** was found to inhibit the attachment of MDA-MB-231 cells to fibronectin, with these effects being mediated by the integrin/FAK and ERK pathways [50]. Compound **8** has been demonstrated to inhibit VEGF-induced angiogenesis by targeting the VEGFR-2 signaling pathway [51]. Treatment with compound **8** caused decreased expression levels of TIMP-1, TIMP-2, VEGF, bFGF, MMP-2, VEGFR-2 and VEGFR-3 in HUVEC [51]. These proteins are closely associated with the metastasis of cancer. Additionally, in MCF-7 cells, compound **8** can diminish the expression of VEGF and HIF-1**α** by blocking the PI3K/Akt/mTOR signaling pathway [28]. Animal studies have also been conducted to explore the potential in vivo therapeutic efficacy of compound **8**. In a MCF-7 xenografted mouse model, it was able to significantly suppress tumor growth and angiogenesis by inhibiting the VEGFR-2 signaling pathway [51]. 

## 5. Conclusions and Future Perspectives

In this review, we aimed to highlight 24 cytotoxic diterpenoids from *E. fischeriana,* which have been the subject of relatively little research, and yet have been shown to be effective agianst numerous cancer types. Emerging anticancer active diterpenoids can be divided into 8 types—*ent*-abietane, daphnane, tigliane, ingenane, *ent*-atisane, *ent*-rosane, *ent*-kaurane, and lathyrane—according to their carbon skeleton and substituents. The antineoplastic mechanisms of these diterpenoids generally include modulation of apoptosis, arresting the cell cycle at various checkpoints, and inhibiting tumor cell metastasis by interfering with multiple signaling pathways. These chemical, structural and molecular approaches represent the basis for more advanced reseach on these anticancer active diterpenoids.

The chemical structure characteristics may affect the anticancer activities of a compound, such as the kind and position of substituents and the linker-chain length [52,53,54]. Hence, structural modifications on the eight subtypes of cytotoxic diterpenoids will become a research topic. This article can serve as a reference for researchers studying their variable relationships between skeleton structure and anticancer activities in order to design novel, highly effective, low-toxicity diterpenoids.

Cancer stem cells (CSCs) are characterized by self-renewal, marked proliferation, and multilineage differentiation [54,55,56,57]. They have been proved to be the vital factor of malignant tumor recurrence and metastasis [54,55,56,57]. The theory of CSCs offers a new target and orientation for tumor therapy. The stem-targeted efficacy and mechanism of Compounds **1**, **10**, **11**, **15**–**24** will be verified in future study.

Recent findings: Nitric Oxide plays an important role in the occurrence and development of tumors [58,59]. In conditions where NO is at lower concentrations (<500 nM), it aids in angiogenesis [58,60]. On the contrary, higher levels of NO (<500 nM) tend to be cytotoxic to cancer cells [58,60]. Therefore, NO has become a new target in tumor treatment. Compounds **1**, **3**, **23**, **24** exhibited promising inhibitory effects on NO production in LPS-induced RAW 264.7 macrophages [61,62]. However, the influence of these diterpenoids on NO production in various cancer cells is still unknown. Investigating the effect of diterpenoids on NO production in cancer cells will help to elucidate the anticancer mechanisms of these compounds.

At present, plant-derived diterpenoids against cancer find it difficult to avoid weak selectivity and toxicity [63,64,65]. A future challenge would be to explore new diterpenoids with high selectivity on the basis of chemical structure diversity. For this reason, these diterpenoids are being continuously tested on normal cells and tissues to evaluate their specificity.

Many diterpenoids mentioned in this article are new diterpenoids isolated from *E. fischeriana*. Therefore, various problems need to be solved. Their cytotoxic activities against a variety of cancer cell lines and their potential molecular mechanisms need to be studied further. More research is essential to exploring how these prodigious molecules interact with the cellular components through molecular chemistry and molecular docking analysis. These would lay the foundation for designing new anticancer drugs with high efficiency and low toxicity.

## Figures and Tables

**Table 1:** Emerging cytotoxic diterpenoids in *E. fischeriana* in vitro.

No.	Compound	Subtype	Type of Cancer	Cell Lines ((IC_50_)	Ref.
**1**	jolkinolide B	*ent*-abietane	liver	HepG-2 (24.43 μM/48 h)	[16]
			breast	MCF-7 (22.76 μM/48 h)	[16,17]
			breast	MDA-MB-231	[18]
			gastric	SGC-7901 (31.32 μM/48 h)	[16]
			gastric	BGC-823 (32.45 μM/48 h)	[16]
			gastric	MGC-803 (34.7 μM/48 h)	[16]
			cervical	Hela (23.12 μM/48 h)	[16]
			human leukemic	U937	[19]
			human leukemic	K562 (12.1 μg/mL/24 h)	[20]
			human leukemic	K562 (11.3 μg/mL/48 h)	[20]
			human leukemic	K562 (10.7 μg/mL/72 h)	[20]
			human leukemic	THP-1	[21]
			human leukemic	HL-60	[21]
			lung	A549 (28.24 μM)	[22]
			melanoma	B16F10	[23]
			prostate	LNCaP (40 μM/48 h)	[24]
			prostate	DU145 (145 μM/48 h)	[24]
			prostate	PC3 (244 μM/48 h)	[24]
**2**	jolkinolide A	*ent*-abietane	liver	HepG-2 (80.12 μM/48 h)	[16]
			breast	MCF-7 (56.34 μM/48 h)	[16]
			lung	A549	[22]
			gastric	SGC-7901 (>100 μM/48 h)	[16]
			gastric	BGC-823 (>100 μM/48 h)	[16]
			gastric	MGC-803 (>100 μM/48 h)	[16]
			cervical	Hela (>100 μM/48 h)	[16]
**3**	17-hydroxyjolkinolide B	*ent*-abietane	liver	HepG-2 (42.13 μM/48 h)	[16]
			breast	MCF-7 (25.33 μM/48 h)	[16]
			gastric	SGC-7901 (44.34 μM/48 h)	[16]
			gastric	BGC-823 (48.12 μM/48 h)	[16]
			gastric	MGC-803 (43.89 μM/48 h)	[16]
			cervical	Hela (35.11 μM/48 h)	[16]
			lung	H460	[16]
			ovary	Skov3	[25]
			colo	Colo205	[25]
			breast	MDA-MB-453	[25]
			breast	MDA-MB-231	[25]
			breast	MDA_MB-468	[25]
			cervix	Hela	[25]
			liver	HepG2	[25]
			blood	Jurkat	[25]
			blood	U937	[25]
			blood	THP-1	[25]
**4**	17-acetoxyjolkinolide B	*ent*-abietane	blood	U937 (0.74 μM)	[26]
			blood	Jurkat (1.06 μM)	[26]
			colon	Colo205 (2.34 μM)	[26]
			gastric	HGC (3.64 μM))	[26]
			breast	MCF-7 (8.74 μM))	[26]
**5**	prostratin	tigliane	liver	HepG-2 (11.77 μM/48 h)	[27]
			breast	MCF-7 (17.4 μM/48 h)	[27]
			gastric	SGC-7901 (25.4 μM)	[27]
**6**	langduin A	daphnane	liver	HepG-2 (35 μM/48 h)	[27]
			breast	MCF-7 (19.4 μM/48 h)	[27]
			gastric	SGC-7901 (21.3μM/48 h)	[27]
**7**	13-*O*-acetylphorbol	tigliane	liver	HepG-2 (32.3 μM/48 h)	[27]
			breast	MCF-7 (18.1 μM/48 h)	[27]
			gastric	SGC-7901 (24.91 μM/48 h)	[27]
**8**	12-deoxyphorbol 13-palmitate	tigliane	breast	MCF-7	[28]
			gastric	BGC823	[29]
			liver	Hep-3B (12.01 μM)	[30]
			lung	A549 (9.38 μM)	[30]
**9**	ingenol-6,7-epoxy-3-tetradecanoate	ingenane	lung	A549 (3.35 μg/mL/72 h)	[31]
			liver	BEL7402 (13.05 μg/mL/72 h)	[31]
			colon	HCT116 (14.62 μg/mL/72 h)	[31]
			breast	MDA-MB-231 (14.42 μg/mL/72 h)	[31]
**10**	ingenol-3-myristinate	ingenane	lung	A549 (2.85 μg/mL/72 h)	[31]
			liver	BEL7402 (15.72 μg/mL/72 h)	[31]
			colon	HCT116 (16.05 μg/mL/72 h)	[31]
			breast	MDA-MB-231 (18.91μg/mL/72 h)	[31]
**11**	ingenol 3-palmitate	ingenane	lung	A549 (2.88 μg/mL/72 h)	[31]
			liver	BEL7402 (25.87 μg/mL/72 h)	[31]
			colon	HCT116 (14.38 μg/mL/72 h)	[31]
			breast	MDA-MB-231 (22 μg/mL/72 h)	[31]
**12**	*ent*-1β,3β,16β, 17-tetrahydroxyatisane	*ent*-atisane	breast	MCF-7 (23.21 μM)	[32]
**13**	*ent*-1β,3α,16β, 17-tetrahydroxyatisane	*ent*-atisane	breast	MCF-7 (15.42 μM)	[32]
**14**	*ent*-kaurane-3-oxo-16β, 17-acetonide	*ent*-kaurane	liver	Hep-3B (8.15 μM)	[30]

**Table 2:** Diterpenoids from *E. fischeriana* inhibiting mammosphere formation in MCF-7 cells.

No.	Bioactive Ingredient	Subtype	Ref.
**1**	jolkinolide b	*ent*-abietane	[14,15]
**10**	ingenol-3-myristinate	ingenane	
**11**	ingenol-3-palmitate	ingenane	
**15**	euphorin E	*ent*-abietane	
**16**	euphorin H	*ent*-abietane	
**17**	yuexiandajisu E	*ent*-abietane	
**18**	ingenol-20-myristinate	ingenane	
**19**	*ent*-3β-hydroxyatis-16-ene-2,14-dione	*ent*-atisane	
**20**	19-*O*-β-d-glucopyranosyl-*ent*-atis-16-ene-3,14-dione	*ent*-atisane	
**21**	euphorin C	*ent*-rosane	
**22**	ebractenoid C	*ent*-rosane	
**23**	ebractenoid F	*ent*-rosane	
**24**	jolkinol A	lathyrane	

**Table 3:** Summary of the anticancer activities of diterpenoids in vivo.

Animal Models	Drug Dose	Conclusions	Ref.
MCF-7 cells xenograft in nude mice	four groups: the negative control group, the jolkinolide B group (40 mg/kg), the 5-Fu group (5 mg/kg), and the jolkinolide B+5-Fu group for 28 days	tumor volume and weight in the 5-Fu, the 5-Fu + jolkinolide B and the jolkinolide B group were greatly reduced, while tumors in the control group reached 1207 mm. However, no significant difference was observed between the JB and the JB+5-Fu group	[17]
B16F10 cells xenograft in C57BL/6 mice	10, 20 and 40 mg/kg of jolkinolide B by intragastric administration for 7 days	The tumor growth inhibition rates were 17.3%, 34.6% and 54.4% in JB-treated groups (10, 20 and 40 mg/Kg)	[23]
BGC823 cells in Female Balb/c nude mice	12-deoxyphorbol 13-palmitate (40 mg/kg) was administered intraperitoneally every three days for two months	tumor growth was significantly suppressed in the 40 mg/mL group compared to the control group	[29]

**Table 4:** Mechanisms of diterpenoids in inducing apoptosis.

No.	Bioactive Ingredient	Type of Cancer	Cell Lines	Mechanisms of Action	Ref.
**1**	jolkinolide B	breast	MDA-MB-231	suppression of the PI3K/Akt signaling pathway	[18]
		human leukemic	U937	suppression of PI3K/Akt and XIAP pathways. cIAP1/2 ↓, Survivin ↓ XIAP ↓ expression Smac ↑ expression activation of caspase-3 and -9.	[19]
			HL-60 THP-1	suppression of the JAK2/STAT3 signaling pathway ↓ JAK2/STAT3 and bcl-2 expression ↑ Bax and cytosolic cytochrome c triggering of caspase-3, -8 and-9 activation	[21]
		mouse melanoma	B16F10	inhibition of glycolysis ↓ mRNA expression of glucose transporter genes (Glut1, Glut3 and Glut4) and glycolysis-related kinase gene(Hk2 and Ldha) ↑ ROS level decreased the potential of mitochondrial membrane	[23]
**3**	17-hydroxyjolkinolide B	Liver Breast breast	HepG2 MDA-MB-231 MDA_MB-468	inhibit STAT3 activation by direct inhibition of JAK kinase activity through covalent crosslinking of the JAKs	[25]
**4**	17-acetoxyjolkinolide B	Liver Cervical lung	HepG2 Hela A549	a inhibitor of IKK inhibit tumor NF-KB activation	[26]
**8**	12-deoxyphorbol 13-palmitate	gastric	BGC823	activation of caspase-3 and -9.	[29]

**Table 5:** Effects of diterpenoids on cell cycles.

No	Bioactive Ingredient	Type of Cancer	Cell Lines	Effects of Diterpenoids on Cell Cycle	Ref.
**1**	jolkinolide B	human leukemic	K562	Cell cycle arrest at G1	[20]
		prostate	LNCap	Cell cycle arrest at G1	[24]
**8**	12-deoxyphorbol 13-palmitate	gastric	BGC823	cell cycle arrest at G2-M checkpoint ↓ cdc2/cyclin B, cyclin A and p-chk1 protein expression	[29]
